# The Impact of Chronic Heart Failure on Retinal Vessel Density Assessed by Optical Coherence Tomography Angiography in Children with Dilated Cardiomyopathy

**DOI:** 10.3390/jcm10122659

**Published:** 2021-06-16

**Authors:** Klaudia Rakusiewicz, Krystyna Kanigowska, Wojciech Hautz, Lidia Ziółkowska

**Affiliations:** 1 Department of Ophthalmology, Children’s Memorial Health Institute, 04-730 Warsaw, Poland; k.kanigowska@ipczd.pl (K.K.); w.hautz@ipczd.pl (W.H.); 2 Department of Cardiology, Children’s Memorial Health Institute, 04-730 Warsaw, Poland; l.ziolkowska@ipczd.pl

**Keywords:** retinal vessel density, dilated cardiomyopathy, optical coherence tomography angiography, foveal avascular zone, retinal thickness

## Abstract

(1) Introduction: The aim of this study is to assess retinal vessel density (VD) in the superficial capillary plexus layer (SP) and deep capillary plexus layer (DP) in children with chronic heart failure (CHF) in the course of dilated cardiomyopathy (DCM) using optical coherence tomography angiography (OCTA). (2) Methods: Thirty children with CHF due to DCM lasting more than six months, with an enlarged left ventricle and impaired left ventricular systolic function (left ventricular ejection fraction (LVEF) ≤ 55%), were enrolled to have both their eyes assessed for this study. Mean age of the children was 9.9 ± 3.57 years. The control group consisted of an additional 30 children without CHF (mean age 11.27 ± 3.33 years) matched for age and gender against the study group. All participants underwent transthoracic echocardiography to measure LVEF using Simpson method. Blood serum was tested for N-terminal-pro-brain natriuretic peptide (NT-proBNP) marker value. All children underwent OCTA with evaluation of the foveal avascular zone (FAZ), whole superficial vessel density (wsVD), foveal superficial vessel density (fsVD), parafoveal superficial vessel density (psVD), whole deep vessel density (wdVD), foveal deep vessel density (fdVD), parafoveal deep vessel density (pdVD), whole thickness (WT), foveal thickness (FT), and parafoveal thickness (PFT). (3) Results: Retinal VD in SP was significantly lower in children with CHF as compared to the controls. The following SP parameters in the study group were statistically significantly lower than these same measurements for the control group. Details, with study group findings quantified first, include wsVD (46.2% vs. 49.83%, *p* < 0.05), fsVD (18.07% vs. 24.15%, *p* < 0.05), and psVD (49.24% vs. 52.51%, *p* < 0.05). The WT (311.03 micrometers (μm) vs. 323.55 μm, *p* < 0.05), FT (244.57 μm vs. 256.98 μm, *p* < 0.05), and PFT (320.63 μm vs. 332.02 μm, *p* < 0.05). No significant differences in DP retinal VD were found between the two groups. No statistically significant differences in the FAZ were found. The fsVD and FT were correlated with biometry and the age of the study participants. There was a correlation between FAZ and FT (*p* < 0.001). There were no correlations between retinal VD in both plexuses and refractive error, sex, NT-proBNP, and LVEF. (4) Conclusions: In children with CHF in the course of DCM as compared to the control group, significantly decreased retinal VD in SP was observed. The results of our study indicate that measurements of the OCTA may be a useful diagnostic method in children with chronic heart failure, but it is necessary to conduct further studies in larger groups of participants and long-term observation of these patients.

## 1. Introduction

Dilated cardiomyopathy (DCM) is defined as left ventricular (LV) dilatation and systolic dysfunction in the absence of underlying abnormal load conditions or coronary artery abnormalities [1,2]. This disease is the most common pediatric cardiomyopathy, with a reported annual incidence of 0.58 to 0.78 cases per 100,000 children [3,4]. Epidemiological studies have reported that DCM is diagnosed in most children under the age of one year, and as many as 93% have features of congestive heart failure [3]. Dilated cardiomyopathy is a significant cause of acute and chronic heart failure, sudden cardiac death, and is the most common indication for heart transplants within the pediatric population [5,6,7]. The main symptoms of DCM are LV dilatation and impairment of systolic function [2,8]. As a result, the most important myocardial function (pumping blood throughout the circulatory system and to individual organs) becomes impaired. In response to reduction of the LVEF and arterial pressure, individual organs and tissues, including the eye and its structures, receive an insufficient supply of oxygen and nutrients in relation to demand [1,8].

The retina consists of ten distinct anatomical layers, some with extremely high metabolic rates, and is supplied with blood from two sources [9,10,11]. The inner layers of the retina are supplied by capillaries from the central retinal artery that arises directly from the opthalmic artery, while the outer layers of the retina are mainly non-vascular and take up oxygen and nutrients from the choroid [9,11]. A network of capillaries consisting of large and medium-sized vessels located in a ganglion cell layer creates a superficial capillary plexus (SP). The deep capillary plexus (DP) is formed by two vascular layers present on each side of the inner nuclear layer. The outer retina in a healthy eye remains completely avascular [12,13]. Within the fovea, there is an area without retinal blood vessels known as the foveal avascular zone (FAZ), with a diameter of approximately 0.5 mm but showing significant differences in size among healthy eyes. The geometric center of the FAZ is often considered the center of the fovea. The branches of vessels belonging to the SP form regular networks around the fovea vascular zone [12,13].

Since the vessels of the fundus of the eye are approximately the same size as the coronary microcirculation and have the same physiological and embryological characteristics, they can serve as representative of the processes occurring in heart diseases [11,14,15]. Several authors note the connection between the vessels of the eye and the heart [11,14,16].

Due to the unique structure of the eyeball, the retina is the convenient place in the human body where blood vessels can be non-invasively observed and analyzed using specific diagnostic techniques [17]. The fundus examination is the basic, standard, routine testing method that allows assessment of changes in blood vessels in the human body; has been used for a long time; and does not require specialized equipment [17]. A common method considered to be the gold standard for precise assessment of retinal blood vessels is fluorescein angiography (FFA). FFA enables dynamic visualization of changes occurring in the retinal vessels; therefore, it is possible to assess severity of exudates, vessel refilling rate, and the area of leakage [18,19,20]. In 2016, optical coherence tomography angiography (OCTA) was presented as a new and promising method of imaging the retinal vasculature. OCTA scans are acquired by analysis of light reflected by vascular and neurosensory tissues of the retina [21,22]. This method is based on detection of erythrocyte movement in the lumen of blood vessels. Tidal signal variations caused by particle movement in a sequence of scans generate contrast, which is subsequently used to generate an image of the retinal vasculature [21,22,23]. The method provides numerical data concerning perfusion in the blood vessels and avascular zones in the imaged retinal area [23]. Blood vessel density (VD) is defined as the percentage of blood vessel surface area in which erythrocyte movement is detectable as compared to the total field of observation [24]. The main advantages of OCTA are the absence of contrast agent and its short test time. In the examination, all layers of the retina are visualized simultaneously and can be analyzed separately. None of the previously used diagnostic methods, including fundus examination and FFA, were able to provide data on the depth of vascular lesions. OCTA allows for a more accurate assessment of avascular zones and pathological structure of vessels that are masked by contrast leakage in later phases of FFA [22,23]. Ji et al. [25], from the basis of their own research, investigated that the position of the scanning beam and resultant scan tilting did not impact the results of measuring the VD in the SP and DP. Therefore, it can be concluded that this method is reliable and repeatable despite the differences in the scanning method and tilt angle of the scan.

In cardiology, the OCTA method can reveal in unprecedented detail the structure of coronary arteries, allowing for a more extensive characterization of coronary artery disease. Optical coherence tomography angiography can examine plaque, such as fibrous tissue, calcium, and lipids, with great accuracy. Due to its ability to distinguish a fresh thrombus from other tissues, it can also identify lesions responsible for the cause of acute coronary syndrome [26]. To our knowledge, this is the first study to use OCTA in patients with CHF due to DCM in the pediatric population.

The primary focus of our study was to assess FAZ, retinal VD in SP and DP, and to determine whether changes in systemic circulation observed in CHF affect retinal blood vessels. The secondary goal was to determine if the VD parameters obtained by OCTA can be used to evaluate the degree of heart failure in children with DCM.

## 2. Material and Methods

This cross-sectional study was conducted at the Department of Ophthalmology and Department of Cardiology, Children’s Memorial Health Institute in Warsaw, between September 2019 and May 2020. It adhered to the tenets of the Declaration of Helsinki and was approved by the Bioethics Committee of the Children’s Memorial Health Institute. All participants 16 years of age and older and the legal guardian(s) of those younger than 16 were provided explanations as to the nature and possible consequences of the study before providing their written informed consent to participate in the study. A total of 60 eyes of 30 children (16 male (M), 14 female (F); mean age 9.9 years ± 3.57; range 5 to 17) with CHF due to DCM were enrolled. The inclusion criteria were patients with CHF lasting for longer than six months with LVEF ≤ 55%. The study group included children with primary DCM; approximately half of them had molecular etiology confirmed using the next-generation sequencing technique (NGS). The control group consisted of 60 eyes of 30 healthy children without diagnosed heart failure or other systemic or ocular disease and was matched for sex (16 M, 14 F) and age (mean age 11.27 ± 3.33; range 4 to 17). The control group consisted of patients consulted for the first time at the ophthalmology clinic, in whom ophthalmologic diseases and refractive error were excluded. The exclusion criteria in both groups included ocular diseases, such as hereditary retinal dystrophy, glaucoma, uveitis, vitreoretinal diseases, previous ocular trauma, retinal laser photocoagulation, eye surgery, significant refractive error (spherical refractive error > ±3 diopter (D), cylindrical refractive error > ±3 D), significant systemic comorbidities, and all other diseases with proven effect on retinal VD. Additionally, scans of low quality were excluded.

Clinical parameters analyzed in children with DCM included serum value of NT-proBNP and LVEF measured in two-dimensional transthoracic echocardiography. Each study participant underwent a full ophthalmic assessment, including best-corrected visual acuity assessed with Snellen’s chart, anterior segment slit lamp biomicroscopy, fundus examination, ocular axial length measurement, and cycloplegic refraction using 1% Tropicamide.

**Figure 1:**
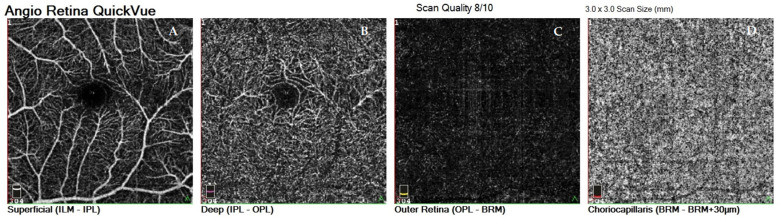
Four vascular layers of the scanned areas of the retina: superficial capillary plexus layer (SP), deep capillary plexus layer (DP), outer retina layer, and choriocapillaries. (**A**) superficial capillary plexus layer, (**B**) deep capillary plexus layer, (**C**) outer retina layer, (**D**) choriocapillaries.

**Figure 2:**
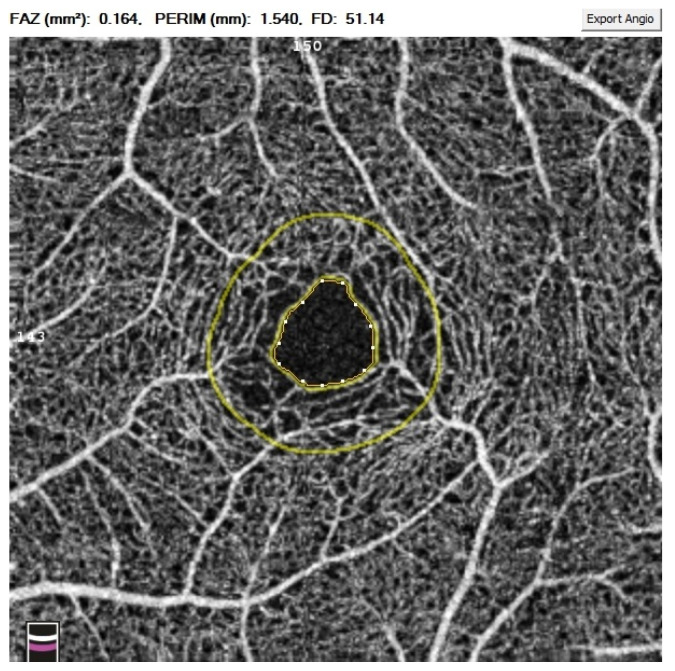
Foveal avascular zone (FAZ) automatically calculated for the superficial capillary plexus.

**Figure 3:**
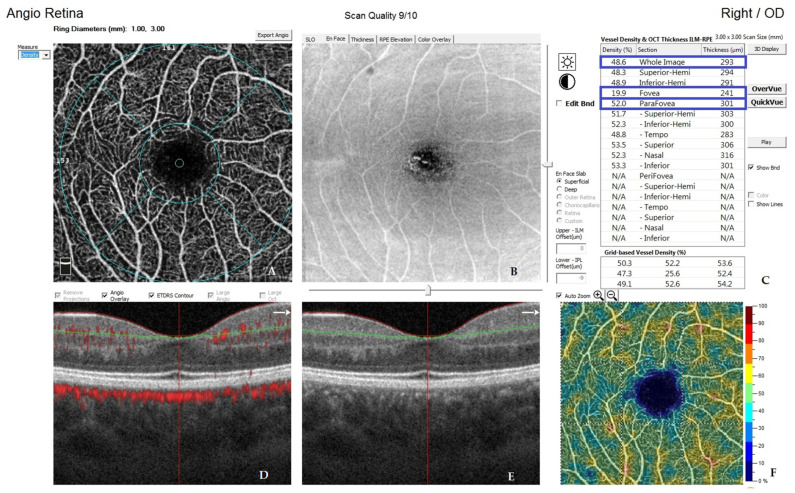
Image presenting automatic calculations of retinal vessel density. The parameters taken for analysis are marked in blue. (**A**) graphical representation of the ring diameter to denote the foveal and parafoveal areas, (**B**) en face angio scan of superficial capillary plexus, (**C**) numerical representation of the results obtained, (**D**) horizontal section through the retina, (**E**) horizontal section through the retina, (**F**) graphical representation of the density of the retinal vessels.

**Figure 4:**
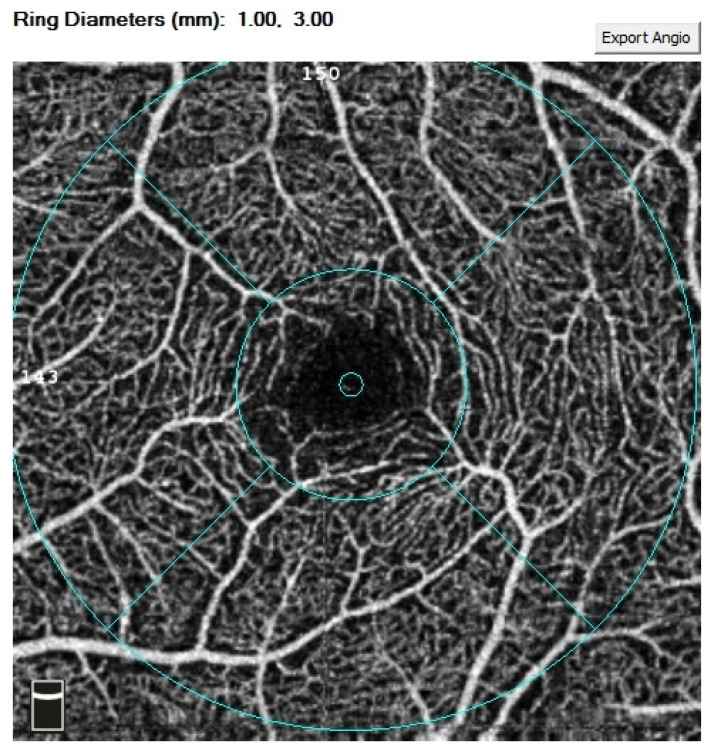
Diagram of tested zones: foveal vessel density and parafoveal vessel density.

All OCTA scans were performed with RTVue-XR (Angiovue; Optovue Inc., Fremont, CA, USA). Scans of a 3 mm × 3 mm area of the macula centered on the fovea were performed in high resolution mode. AngioVue software automatically separates the scanned area into four layers: the SP, DP, outer retina layer, and choriocapillaries (Figure 1). For analysis, the parameters of the SP and DP were of primary use. The retinal VD was calculated as a percentage of the area occupied by flowing blood vessels in the selected area. To quantify the FAZ area (mm^2^) and macular VD (%), the software’s pre-programmed algorithms were used. The FAZ area was calculated automatically for the SP (Figure 2). Measurements taken in the SP included whole superficial vessel density (wsVD), foveal superficial vessel density (fsVD), and parafoveal superficial vessel density (psVD). Measurements of the DP included whole deep vessel density (wdVD), foveal deep vessel density (fdVD), and parafoveal deep vessel density (pdVD) (Figure 3). The foveal region was a 1-mm circle with a hole in the center. The foveal region was surrounded by the parafoveal region, a ring with an internal diameter of 1 mm and external diameter of 3 mm; Figure 4 diagrams this structure. Data on foveal thickness (FT) and parafoveal thickness (PFT) measured in micrometers (μm) were obtained from retinal maps using the same device. Head stabilization was achieved by standard support of chin and forehead. Study participants were instructed to focus on the internal fixation target. Scans of low quality, with artifacts, or blurred motion for which data were insufficient for proper analysis were excluded. If initial scan quality was poor, the test was repeated until the image quality met the requirements. Data collected from both eyes of the individual were analyzed [27].

## 3. Statistical Analysis

Statistical analysis was performed using R 3.5.1 software (R Core Team (2018)). The analysed variables were expressed as means, standard deviations, 95% confidence intervals, and ranges. The Mann–Whitney two-way test for two independent samples was applied to determine the presence of statistical differences between the study group and the controls. Linear relationships between selected quantitative variables were assessed using the Pearson correlation coefficient. The level *p* < 0.05 was considered statistically significant [26].

## 4. Results

Both eyes of 30 study participants (16 M, 14 F), all diagnosed with CHF due to DCM, were measured and compared with a control group consisting of 30 matched children with no heart disease (16 M, 14 F). Children in the study group were a mean age of 9.9 ± 3.57 years (range 5 to 17 years). Children in the control group were 11.27 ± 3.33 years (range 4 to 17 years, *p* = 0.11). Clinical characteristics of both groups are presented in Table 1. Demographic data, including age and gender, were comparable in both groups. The mean value of LVEF in the DCM group was 49.03% (range 30% to 55%); the mean value of NT-proBNP was 568.1 pg/mL (range 15 to 3723 pg/mL). All enrolled participants had full visual acuity (20/20) as assessed using the Snellen chart.

**Figure 5:**
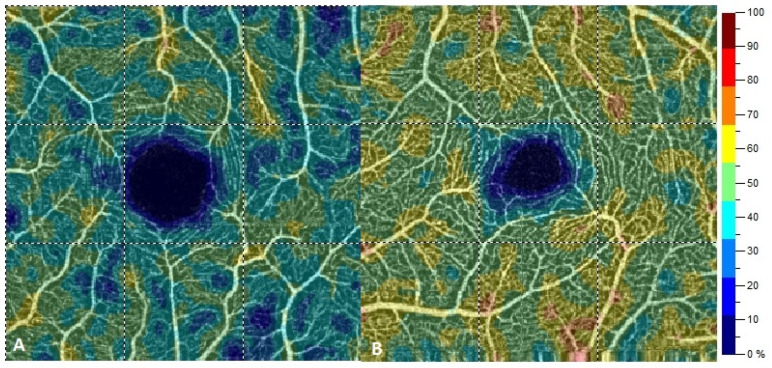
Scans of retinal vessel density in superficial capillary plexus in the group with dilated cardiomyopathy (**A**) and in the control group (**B**).

**Figure 6:**
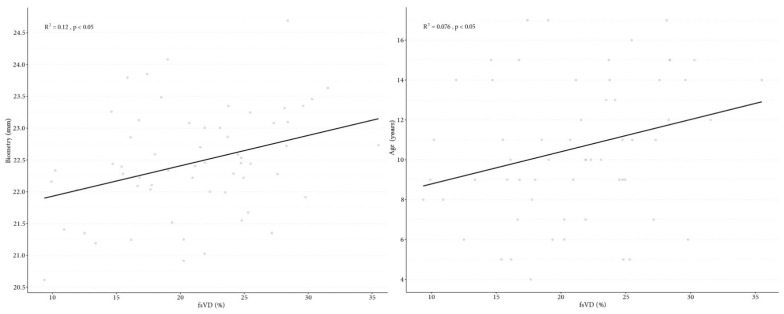
Correlation graph of foveal vessel density in superficial capillary plexus to biometry (**A**) and correlation graph of foveal vessel density in superficial capillary plexus age (**B**).

**Figure 7:**
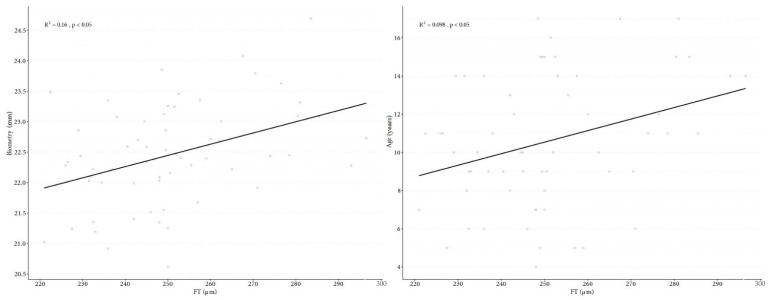
Correlation graph of fovea thickness to biometry (**A**), correlation graph of fovea thickness and age (**B**).

Retinal VD parameters, such as wsVD, fsVD, and psVD, were significantly lower in the SP of DCM patients than in the control group (Figure 5). Parameter differences in SP between the study and the control group included: wsVD (46.2% vs. 49.83%, *p* < 0.05), fsVD (18.07% vs. 24.15%, *p* < 0.05), and psVD (49.24% vs. 52.51%, *p* < 0.05). The following measurements were significantly thinner in children with DCM than those in the control group: whole thickness (WT) (311.03 μm in the study group vs. 323.55 μm in the controls, *p* < 0.05), FT (244.57 vs. 256.98 μm, *p* < 0.05), and PFT (320.63 μm vs. 332.02 μm, *p* < 0.05). No significant differences in VD in the DP were found between the two groups. Additional measurement differences between the two groups include wdVD (49.7% in the study group vs. 50.04% in controls, *p* = 0.55), fdVD (33.94% vs. 36.62%, respectively, *p* = 0.08), and pdVD (52.13% vs. 51.85%, *p* = 0.29). There were no significant differences in the FAZ between the groups (0.27 mm^2^ in the study group vs. 0.24 mm^2^ in the controls, *p* = 0.2). Table 2 provides detail data. Foveal VD in SP correlated with biometry (*p* = 0.008) and study participant age (*p* = 0.033) (Figure 6), and FT correlated with biometry (*p* = 0.002) and the age of patients (*p* = 0.015) (Figure 7). Correlation was found between FAZ and FT (*p* < 0.001). In contrast, no correlations were found between FAZ, WT, FT, PFT, VD in SP and DP, value of NT-proBNP, and LVEF.

## 5. Discussion

DCM is characterized by LV systolic dysfunction with concomitant tissue remodeling leading to heart failure [28]. It is the most common type of cardiomyopathy in children [3,4]. Genetic, viral, immunological, metabolic, and cytotoxic factors influence etiology, although cause remains unknown (idiopathic DCM) in almost 50% of cases [2]. In symptomatic patients, gradual disease progression is observed, and the mortality rate within one to five years from onset of clinical symptoms is approximately 30% [4]. LV systolic dysfunction is a key pathophysiological aspect in development and progression of CHF [8]. The reduction of LVEF and arterial pressure cause an insufficient supply of oxygen that can lead to hypoxia of all organs and tissues of the body, including the eye and its structures [1,8].

Retinal blood vessels are routinely assessed when diagnosing systemic diseases that can include diabetes, hypertension, anemia, and heart failure; this eye examination reveals a relationship between systemic blood vessels and retinal microvasculature that has proven highly valuable for diagnosing a host of medical conditions [11,14,16,29,30,31,32]. Medical researchers have long been interested in the correlation between the condition of the retinal blood vessels, their caliber and size, and risk for cardiovascular diseases, including assessment of the risk of cardiac death [32,33]. Some authors emphasize the particular importance of assessing retinal blood vessels in cardiac diseases [14,16,34]. Witt et al. [35] noted a correlation between the diameter and tortuosity of retinal arteries and the risk of death from ischemic heart disease. Others reported correlations of retinal microvascular signs with LV hypertrophy [36] and extent of LV concentric remodeling [37]. Others observed the presence of cardiovascular risk factors in patients with changes to retinal blood vessels [38,39,40,41].

This study is thought to be the first to use OCTA to assess changes in retinal VD in children with CHF due to DCM. Previous studies using other diagnostic methods to assess health of retinal vessels in patients with heart failure are available for review; these studies also indicate the existence of significant damage to retinal blood vessels associated with circulatory disturbances caused by heart failure.

Phan et al. [42] found a relationship between diameter of the retinal blood vessels and heart failure. A computer-assisted program assessed photographs of the fundus to determine caliber of retinal blood vessels. Retinal arterioles of wider diameter were significantly and independently associated with heart failure; this association was much stronger among participants with diabetes than without. There was no association between the size of the retinal vein and heart failure. Nägele et al. [43] performed retinal vessels analysis using flicker-light-stimulation in adults with CHF. The authors noted a significant reduction in flicker-light-induced dilatation of the arterial and venular vessels of the retina, demonstrating profound changes in retinal microvascular function. Moreover, it was noted that the degree of retinal veins dysfunction correlated with left atrial volume index and inversely correlated with value of NT-proBNP.

With the development of new technologies and research methods, the authors began to analyze the condition of retinal microcirculation using OCTA, based on parameters such as VD [16,30,31,34].

Arnould et al. [44] analyzed retinal VD based on OCTA in 30 patients with myocardial infarction during the acute phase and three months after cardiac rehabilitation. The authors found no significant relationship between retinal vascular density and hemodynamic variables in patients with myocardial infarction. However, Wang et al. [34] examined retinal VD in 158 adult patients with coronary heart disease (CHD) using OCTA. WholeVD and parafovealVD in both superficial and deep plexuses were found to be lower to a statistically significant degree in patients with CHD versus those without it (*p* < 0.001). It should be noted that no differences in fovea VD in SP and DP (*p* = 0.28, *p* = 0.20, respectively) were identified between the groups. Surprisingly, CHD patients were found to have increased wholeVD and parafovealVD in the outer retina relative to individuals without CHD. Additionally, a decrease in wholeVD, foveaVD, and parafovealVD in the choroid capillaries was noted. In contrast, Li et al. [45] analyzed VD in 117 patients with congenital heart disease (CD); 60 of them had acyanotic congenital heart disease (ACD), and 57 had cyanotic congenital heart disease (CCD). The authors noted a significant difference in vascular density: wholeVD (*p* = 0.010) and perifovealVD (*p* = 0.004) in SP in CCD patients compared to the control group. The analysis showed that the mean VD in DP was lower in patients with CCD compared to patients with ACD and compared to the control group (in parafovea and perifovea, both *p* < 0.001). Also of note is the finding that, in CD patients, VD was positively correlated with oxygen saturation (SaO_2_) but negatively with hematocrit. Arnould et al. [46] used OCTA to identify correlations between retinal VD and cardiovascular risk in patients with acute coronary syndrome (ACS) during the EYE-Myocardial Infarction (EYE-MI) pilot study. In a study involving 272 study participants, inner VD in SP for ACS patients was measured at 20.30 mm^−1^ (18.60 to 21.20) and 21.85 mm^−1^ (20.80 to 22.58) for study participants without ACS. This difference, lower in patients with ACS than without it, was proven to be statistically significant. Low retinal VD was associated with low LVEF and high cardiovascular risk as defined by the American Heart Association. Arnould et al. showed that the LVEF was inversely proportional to inner VD (*p* = 0.001). The findings of this current study—that cardiovascular disease can lower vascular density—are in keeping with the findings of the above-referenced studies conducted earlier. It is believed that peripheral vasoconstriction in response to low cardiac output is the pathophysiological mechanism that triggers this outcome [39,43,45]. Almeida–Freitas et al. [47] recorded higher resistance index and lower diastolic velocity in the ocular arteries of patients with low LVEF. The blood supply to the retina is physiologically kept constant due to specific autoregulation mechanisms. The decrease in VD in CHF patients observed in our study can be explained by insufficiency to auto-regulate the retinal blood supply; it may be assumed that it results from the failure of local mechanisms that lead to narrowing and occlusion of the retinal vessels’ end branches.

Hypoxia associated with systemic circulatory insufficiency affects vessels of various caliber in varying degree. Anatomically, the SP consists of large and medium-sized arterioles surrounded by smooth muscle cells, while DP includes capillaries consisting only of endothelium, basement membrane, and pericytes. [48]. Pericytes are more sensitive to hypoxia, making DP vessels more sensitive to circulatory changes and ischemia [49]. In our study, however, we found significantly larger changes in the SP than VD changes in the DP. To date, there is insufficient analysis of abnormalities in the network of retinal vascular plexuses based on OCTA to explain why the changes in the blood vessels of the retina have occurred in this configuration. More research is needed to explain why children with CHF developed changes of greater intensity in the SP than in the DP.

The fovea is a highly specialized macular area that is the central point of the most focused vision with the highest resolution. The histological structure of the fovea is different from that of the rest of the retina, with the central area mainly consisting of cones and a vascular-free area known as the FAZ. The FAZ is surrounded by the terminal ring of the capillary network of retinal arterioles that originate from the superficial capillary plexus [50]. When using OCTA, the contrast it produces between vascular-free and vascular-rich zones allows for visualization of the network of the FAZ area [12]. Based on studies involving healthy adult patients, the mean surface area of FAZ measured with OCTA ranged from 0.24 mm^2^ to 0.48 mm^2^ in SP and from 0.38 mm^2^ to 0.70 mm^2^ in DP [12,51,52,53]. The results of our study demonstrated that FAZ in the SP was 0.27 mm^2^ in children with DCM and 0.24 mm^2^ in those without DCM. In our analysis, there was no statistically significant difference in FAZ parameters between groups. There are few, if any, existing studies that use OCTA to analyze the FAZ surface of CHF patients. To our knowledge, this study is the first to assess the influence of circulatory failure caused by DCM on FAZ. To date, the influence of systemic diseases on the surface of FAZ is a subject of widespread interest. Previous studies have focused on how diabetes mellitus and hypertension affect the surface of FAZ, wherein a significant reduction in the number of vessels in the central area of the fovea was found to produce an increase of surface FAZ [54,55,56]. Additional studies indicate that FAZ correlates with the axial length of the eyeball, gender, and age [52,57]. Other studies did not find similar correlations [13,53]. In our study, we did not reveal any correlations between FAZ and biometry, age, or gender. Tan et al. [52] noted that FAZ in SP and DP differed depending on central retinal thickness; a smaller FAZ was associated with thicker retina. Lupidi et al. [58] discovered a strong negative correlation between FAZ area and thickness and volume of the central retina in both the SP (r = 0.7, *p* < 0.001) and DP (r = 0.69, *p* < 0.001). Other studies reached similar conclusions. When FT and FAZ area were compared in our study, a correlation between FT and FAZ (r = 0.461, *p* < 0.001) was noted.

In this particular study, children with DCM had a significant decrease in FT and PFT compared to the control group. These results align with similar studies covering cardiovascular diseases. Wang et al. [34] noted the mean retinal thicknesses in patients with CHD was lower in all eight regions than in the control group, but these differences were not statistically significant. In the group of CHD patients compared to the control, FT was 239.2 μm versus 242 μm (*p* = 0.2), and PFT was 307 μm versus 311 μm (*p* = 0.28). De Aguiar Remigio et al. [59] reported significant reduction in FT (187.0 ± 4.4 μm vs. 236.9 ± 2.7 μm, *p* < 0.001) in children with cyanotic congenital heart disease compared to controls. In contrast, Aydi et al. [60] found no reduction in central macular thickness in patients with cardiovascular risk factors that included previous acute myocardial infarction, acute coronary syndrome, coronary revascularization (with a stent or coronary artery bypass graft), or significant plaque on coronary angiography. Reduction in retinal thickness may reflect damage caused by low oxygen supply, as hemodynamic regulation is essential for the structural and functional integrity of the retina. Based on numerous studies of healthy volunteers, it was calculated that the mean central fovea thickness ranges from 182 μm to 253.92 μm [61,62,63,64,65]. Some studies found correlation between FT and axial length of the eyeball, age, and refractive error [63]; however, no such correlations were identified in other studies [62,63,64,65]. A correlation between FT and biometrics as well as patient age were revealed in own study.

In our study, we found no correlation between VD in the SP and DP and cardiac parameters, such as NT-proBNP and LVEF. It can be assumed that this is due to the different dynamics of changes in individual parameters, which do not interact directly. This issue and the correlation of cardiac parameters with density of the retinal vessels require further, more detailed analyzes.

The main advantage of our study is its use of a non-invasive test that provides repeatable and quantitative evaluation of abnormalities in retinal VD in a homogeneous group of CHF patients. This study does come, however, with several limitations. The total study group was not very large, prompting the need for further studies on larger numbers of study participants. The influence of individually started treatment of heart failure in children with DCM on the obtained results of retinal VD cannot be excluded. Retinal VD was assessed in the area of the central retina measuring 3 mm × 3 mm; therefore, not all retinal vessels, including those in the periphery, were assessed.

## 6. Conclusions

In children with chronic heart failure and dilated cardiomyopathy, optical coherence tomography angiography demonstrated a reduction in retinal VD in the superficial capillary plexus, FT, and PFT. CHF can impair the late retinal remodeling that occurs during the first few years of life. Further long-term observation in a larger number of patients will determine if this has any visual consequences and whether the method may be useful in clinical practice in children with DCM.

## Figures and Tables

**Table 1:** Clinical characteristics of both groups.

Variable	Study Group	M	SD	95% CI	Range	*p*
NT-proBNP (pg/mL)	DCM Group	568.10	1045.27	177.79–958.41	15–3723	-
LVEF (%)	DCM Group	49.03	6.63	46.56–51.51	30–55	-
Age (years)	Control Group	11.27	3.33	10.02–12.51	4–17	0.11
	DCM Group	9.9	3.57	8.57–11.23	5–17	
Biometry (mm)	Control Group	22.75	0.69	22.49–23.01	21.35–24.69	<0.05
	DCM Group	22.17	0.88	21.85–22.5	20.615–24.08	
Spherical refractive error	Control Group	0.12	0.84	−0.19–0.44	−1.75–1.75	
	DCM Group	0.75	1.14	0.32–1.17	−2.00–3.00	
Cylindrical refractive error	Control Group	0.25	0.27	0.15–0.35	0.00–1.00	0.14
	DCM Group	0.17	0.29	0.06–0.28	0.00–1.25	

M, mean; SD, standard deviation; CI, confidence interval; LVEF, left ventricular ejection fraction; NT-proBNP, natriuretic peptide type B.

**Table 2:** Descriptive statistics for the parameters of retinal vessel density, FAZ, and retinal thickness (patients with DCM compared to control).

Variable	Study Group	M	SD	95% CI	Range	*p*
FAZ	Control Group	0.24	0.09	0.21–0.28	0.087–0.445	0.2
	DCM Group	0.27	0.07	0.24–0.3	0.138–0.4795	
wsVD	Control Group	49.83	1.32	49.33–50.32	47.65–53.15	<0.05
	DCM Group	46.2	2.24	45.36–47.04	41.95–52.2	
fsVD	Control Group	24.15	5.18	22.21–26.08	15.4–35.5	
	DCM Group	18.07	5.09	16.16–19.97	9.35–27.3	
psVD	Control Group	52.51	1.25	52.04–52.97	50.15–54.7	
	DCM Group	49.24	2.5	48.31–50.18	43.7–55.75	
WT	Control Group	323.55	13.16	318.64–328.46	294–342	
	DCM Group	311.03	14.74	305.53–316.54	281.5–334	
FT	Control Group	256.98	18.46	250.09–263.88	222.5–296.5	
	DCM Group	244.57	15.56	238.76–250.38	221–285.5	
PFT	Control Group	332.02	15.98	326.05–337.99	291–354.5	
	DCM Group	320.63	15.19	314.96–326.31	292–344	
wdPD	Control Group	50.04	3.71	48.65–51.42	41.05–56.1	0.55
	DCM Group	49.7	2.98	48.58–50.81	44.8–54.4	
fdVD	Control Group	36.62	5.36	34.61–38.62	24.9–45.6	0.08
	DCM Group	33.94	5.37	31.93–35.95	18.95–41.35	
pdVD	Control Group	51.85	3.79	50.44–53.27	42.1–58.55	0.79
	DCM Group	52.13	3.11	50.97–53.29	46.15–56.85	

M, mean; SD, standard deviation; CI, confidence interval; FAZ, foveal avascular zone; wsVD, whole vessel density in superficial capillary plexus; fsVD, foveal vessel density in superficial capillary plexus; pdVD, parafoveal vessel density in superficial capillary plexus; WT, whole thickness; FT, foveal thickness; PFT, parafoveal thickness; wdPD, whole vessel density in deep capillary plexus; fdVP, foveal vessel density in deep capillary plexus; pdVD, parafoveal vessel density in deep capillary plexus.

## Data Availability

The data presented in this study are available on request from the corresponding author. The data are not publicly available due to ethical reasons.
